# Socio‐Demographic Inequalities in COVID‐19 Health Care Access and Experiences in the United Kingdom: Intersectional and Mixed‐Methods Analyses of Open and Closed Questions in a Prospective Cohort Study

**DOI:** 10.1111/hex.70692

**Published:** 2026-06-01

**Authors:** Nathan J. Cheetham, Anoushka Beattie, Alastair B. Comery, Vicky Bowyer, The COVID Symptom Study Biobank Consortium, Michela Antonelli, Julia Brown, Liane Canas, Joan Capdevila Pujol, Lynn Cherkas, Jie Deng, Katie Doores, Emma Duncan, Maria Paz Garcia, Alexander Hammers, Deborah Hart, Nicholas Harvey, Adrian Hopper, Christina Hu, Eric Kerfoot, Michael Malim, Marc Modat, Erika Molteni, Benjamin Murray, Ayrun Nessa, Sebastien Ourselin, Tim Spector, Claire Steves, Carole Sudre, Samuel Wadge, Jonathan Wolf, J. D. Carpentieri, Claire J. Steves

**Affiliations:** ^1^ Department of Twin Research and Genetic Epidemiology King's College London London UK; ^2^ Institute of Education University College London London UK; ^3^ Department of Social Policy Sciences, Centre for Death and Society University of Bath Bath UK; ^4^ Guy's and St Thomas's NHS Foundation Trust London UK; ^5^ School of Biomedical Engineering & Imaging Sciences King's College London London UK; ^6^ James Cook University Townsville Australia; ^7^ ZOE Ltd London UK; ^8^ Department of Infectious Diseases King's College London London UK; ^9^ Department of Endocrinology Guy's and St Thomas' NHS Foundation Trust London UK; ^10^ King's College London and Guy's and St Thomas' PET Centre London UK; ^11^ Department of Ageing and Health Guy's and St Thomas' NHS Foundation Trust London UK; ^12^ MRC Unit for Lifelong Health and Ageing, Department of Population Science and Experimental Medicine University College London London UK; ^13^ Department of Computer Science, Centre for Medical Image Computing University College London London UK

**Keywords:** health care access, intersectionality, long COVID, mixed methods, patient experience, quality of care

## Abstract

**Introduction:**

Inequalities in health care access and experiences during the COVID‐19 pandemic have been observed along axes of social advantage. It is less well understood how access to care varied intersectionally with combinations of multiple social factors, and how social advantage shaped care experiences for COVID‐19 illness.

**Methods:**

We analysed responses to both closed (*N* = 3516) and open (*N* = 335) survey questions relating to health and social care access and experiences during the first two and a half years of the COVID‐19 pandemic in the United Kingdom community‐based cohort, COVID Symptom Study Biobank. Causal effects of individual socio‐demographic variables on access to health and social care were estimated with multivariable regression models, weighted for inverse probability of survey completion and adjusted for potential confounders. Associations between care access issues and social strata comprising combinations of sex, education level and local area deprivation were estimated using the intersectional multilevel analysis of individual heterogeneity and discriminatory accuracy (MAIHDA) approach. Responses to open questions on health care experiences for COVID‐19 illness were deductively coded and quantitatively analysed to estimate associations between socio‐demographic advantage and various aspects of care experiences.

**Results:**

Gradients in health and social care access along the lines of social advantage were observed in intersectional MAIHDA models, with the predicted probability of access issues highest for the stratum comprising female participants with lowest education and highest deprivation levels (42.9%, 95% CI: 31.9%–54.6%), and lowest for male participants with highest education and lowest deprivation (18.7%, 95% CI: 12.8%–26.7%). Socially disadvantaged participants also reported receiving poorer care for COVID‐19, with lower likelihood of reporting receiving adequate care and specialist care for long COVID, and higher likelihood of negative experiences of care versus advantaged participants.

**Conclusions:**

Inequalities in likelihood of health and social care access issues were observed, as well as inequalities in care experiences specifically for COVID‐19, with issues accessing care and poorer experiences more likely to be reported by individuals with greater social disadvantage.

## Patient or Public Contribution

Members of the COVID Symptom Study Biobank Volunteer Advisory Panel provided feedback on the questionnaire that was our primary data source for this analysis. Previous discussions with the Volunteer Advisory Panel informed the aims and objectives of this study.

## 1 Introduction

Disruptions to health and social care services during the COVID‐19 pandemic have been commonly reported in the United Kingdom [1, 2] and have been associated with a lower probability of self‐reported recovery from COVID‐19 [3]. Issues accessing care during the pandemic appear to have been unequally distributed along axes of structural social advantage, with a higher likelihood of disruptions for female versus male sex, racially minoritised versus white ethnic groups, and for those in manual/routine versus managerial/professional occupations. Gradients in health and health outcomes have also been consistently observed over time within the United Kingdom, including in the most recent Census in 2021, along similar axes of advantage, including race/ethnicity [4], education level [5, 6], occupational class [7, 8], income [9], and local area deprivation [10]. Social gradients were also seen for COVID‐19 incidence and mortality [11, 12], and prevalence and recovery from long‐term ongoing symptoms, or long COVID [3, 13, 14], with poorer outcomes for those with greater social disadvantage. Discrepancies between prevalence of long COVID based on self‐report [15, 16, 17], and from coding in electronic health care records (EHR) in the United Kingdom [18, 19, 20], where prevalence is highest for those in the most deprived areas by self‐report, but coded in EHR most often for those in the least deprived areas, as well as greater symptom intensity among more disadvantaged groups within long COVID clinical populations [21], suggests a greater unmet need for long COVID care within more disadvantaged populations. In combination, these reports suggest the presence of the ‘inverse care law’ [22], during the COVID‐19 pandemic, where the availability of health care was associated inversely with health care need.

In qualitative studies of health care access and experiences of those living with long COVID in England, Scotland and Austria, common themes include difficulties getting appointments with general practitioners, struggling to navigate complex health care systems, lack of awareness and knowledge of long COVID from health care providers, anticipated stigma based on minoritised identity factors or stereotyping of long COVID, and negative, often discriminatory, experiences with health care providers [23, 24, 25, 26, 27, 28, 29]. Intersectional inequalities have also been observed during the COVID‐19 pandemic in both access to care [30], and in experiences of care [23, 29], with greater access issues and poorer experiences for women from racially minoritised or immigrant backgrounds. However, no studies have looked at socio‐demographic variation in health care access and experiences using a quantitative intersectional approach. To‐date, outcomes in quantitative studies have been limited to access to health care, and examined socio‐demographic factors as individual exposures only. There have also been no studies that have considered responses to open and closed survey questions in combination.

Given reports of health care access and experiences being shaped by explicit combinations or ‘intersections’ of socio‐demographic factors in smaller qualitative studies, there is a desire for more research to understand whether quantitative, intersectional differences in care access and experiences are replicated within larger samples [31]. Furthermore, understanding socio‐demographic variation in care specifically for COVID‐19 is also particularly crucial, given the known impacts of COVID‐19 and long COVID on health and wider circumstances [32], and the effectiveness of early pharmacological treatment in reducing the likelihood of long COVID [33], and of specialist care programmes taking a cognitive and behavioural approach for those living with long COVID [34]. Therefore, in this study, we use a mixed methods approach to answer the research question: how did pre‐pandemic social advantage and disadvantage affect access to, and experiences of, health and social care during the COVID‐19 pandemic? First, we estimated associations between pre‐pandemic markers of social advantage and disadvantage and health and social care access issues/disruptions during the first 2 years of the COVID‐19 pandemic using self‐reported responses to closed survey questions. We estimated associations with socio‐demographic factors both individually and in combination using an intersectional approach. Second, we qualitatively coded a subset of responses to an open‐text question where participants described experiences of health care specifically for COVID‐19 illness; we then quantitatively analysed how care access and experiences varied according to cumulative social advantage or disadvantage, while accounting for health care need. Quantitative analyses of coded open‐text responses are supported by illustrative quotations. We hypothesised that social strata with combinations of advantaged positions/statuses would be less likely to report experiencing access issues and report better experiences of care, while those with combinations of multiple disadvantaged statuses would be more likely to report access issues and worse care experiences.

## 2 Methods

### 2.1 Data Sources

#### 2.1.1 COVID Symptom Study Biobank

Study participants were volunteers from the COVID Symptom Study Biobank (CSSB) cohort. CSSB participants were recruited via the COVID Symptom Study app from ZOE Ltd (CSS), launched in the United Kingdom on March 24, 2020. All data were collected with informed consent obtained online. The CSS app allowed participants to self‐report demographic information, symptoms potentially suggestive of COVID‐19 infection, any SARS‐CoV‐2 testing and results, and any vaccinations. CSS participants from across the United Kingdom were invited to join the CSSB by email from October to November 2020 and from May to June 2021. Invitation to join the CSSB targeted five groups with different statuses at the time of invitation, derived from CSS data: asymptomatic COVID (positive SARS‐CoV‐2 test and no associated symptoms); ‘short COVID’ (positive SARS‐CoV‐2 test and 1‐13 days of symptoms); ‘long COVID’ (positive SARS‐CoV‐2 test and ≥ 28 days' symptoms); ‘long non‐COVID’ (negative SARS‐CoV‐2 test and ≥ 28 days' symptoms); and ‘healthy non‐COVID’ (negative SARS‐CoV‐2 test and ≤ 3 days with ≤ 3 symptoms). Before the invitation, individuals were matched based on minimum Euclidean distance for age, sex and body mass index (BMI) across groups. The aim of the targeted approach was to give five equally‐sized, matched groups, to allow investigation of the long‐term effects of COVID‐19 on health. As such, cohort composition in terms of prevalence of COVID‐19 and long COVID at the time of recruitment is not representative of the wider UK population.

CSSB participants were invited (*N* = 8324) to participate in the ‘*COVID Reflections ‐ 2 Years On’* online questionnaire in August 2022. Questionnaire data were supplemented with data collected at the time of registration with the CSS app, at consent to CSSB, and in earlier CSSB studies. Variables described below were collected as part of the August 2022 questionnaire unless otherwise stated. The CSSB Volunteer Advisory Panel were consulted on the delivery of the August 2022 questionnaire and gave recommendations on invitation and reminder strategies.

#### 2.1.2 Outcome Variables (Closed Question Analysis): Health and Social Care Access Issues During the COVID‐19 Pandemic

Health and social care access issues during the COVID‐19 pandemic, independent of reason for seeking care, were measured in the August 2022 CSSB questionnaire as part of a more general question on adverse pandemic experiences asked to all questionnaire respondents, ‘*Have you experienced any of the following during the COVID‐19 pandemic?’* Respondents were asked to report Yes/No/Prefer not to answer for the following items: *(a) Lost your job/been unable to do paid work, (b) Been put on furlough from your job (paid leave), (c) Unable to pay bills, (d) Evicted/lost accommodation, (e) Unable to afford food, (f) Unable to access required medication, (g) Unable to access health services in the community, for instance, GP/community physiotherapy/nurse/podiatrist/dentist, (h) Unable to access the community social care services or voluntary sector support you need, for instance, from carers or day centres, (i) Unable to access inpatient or outpatient appointments booked at a hospital for a consultation, investigation, treatment, or surgery, (j) Unable to access appointment for cognitive behaviour therapy, counselling, or psychological therapy, (k) You lost somebody close to you due to COVID‐19, (l) Change in relationship status*. Five response items, *f*, *g*, *h*, *i*, and *j*, were used to derive a continuous variable counting the number of health and social care access issues, and a binary variable indicating the presence of one or more issues.

#### 2.1.3 Outcome Variables (Open Question Analysis): Health Care Experiences for COVID‐19 Illness

Outcome variables describing aspects of health care access and quality, specifically for COVID‐19 illness, were derived from qualitative coding, described in detail further on, of responses to the open‐text question, ‘*Please use the space below to tell us a bit more about any care you received for COVID‐19? For example, was it easy to see your GP? Were there any parts of your treatment that were particularly helpful or that you think could be improved? Please write as much or as little as you like about any aspects of your healthcare experience’*, prefaced with, ‘*We are interested in hearing about your experience with the healthcare system and how those experiences have impacted your health and, if relevant, any COVID‐19 symptoms’.*

To generate variables for quantitative analysis, qualitative coding of open‐text responses was done by two of the authors using a deductive approach, using a predetermined codebook generated based on the research questions of the study (full codebook with unadjusted frequencies in Table S6) [35]. Outcome variables were generated from either single codes or combinations of multiple codes (Table 1).

**Table 1:** Qualitative codes used to define COVID‐19 health care outcomes for quantitative analysis. The full codebook is given in Table S6.

Outcome: Category	Qualitative code(s) description	Code #
Care needed for COVID‐19: Yes	Did not receive care for COVID‐19 ‐> did not attempt to contact health care services ‐> but wanted or needed to	11
	OR	
	Did not receive care for COVID‐19 ‐> Attempted to use health care services but did not receive care	13
	OR	
	Received care for COVID‐19	16
Care needed for COVID‐19: No	Did not receive care for COVID‐19 ‐> did not attempt to contact health care services ‐> and did not need them	12
Care needed for COVID‐19: Unknown	None of the above ‘Care needed for COVID‐19’ codes	
Care received: Yes, inferred as adequate	Received care for COVID‐19 AND NOT coded with any of the below ‘Care received’ codes	16
Care received: Yes, perceived as inadequate	Received care for COVID‐19 ‐> Received care (perceived as) inadequate ‐ care received was unhelpful or irrelevant, had an appointment but effectively no care was provided, etc.	25
Care received: No, did not attempt to access	Did not receive care for COVID‐19 ‐> did not attempt to contact health care services ‐> but wanted or needed to	11
Care received: No, access attempt unsuccessful	Did not receive care for COVID‐19 ‐> Attempted to use health care services but did not receive care	13
Received care from a long COVID clinic or specialist	Received care for COVID‐19 ‐> Received care in ‘long covid clinic’ or from a specialist	19
Ease of care access: Easy	Received care for COVID‐19 ‐> Accessing was easy/straightforward (explicit or inferred)	21
Ease of care access: Difficult	Received care for COVID‐19 ‐> Accessing was difficult (explicit or inferred)	22
Ease of care access: Did not attempt access	Did not receive care for COVID‐19 ‐> did not attempt to contact health care services ‐> but wanted or needed to	11
Ease of care access: Not stated	None of the above ‘Ease of care access’ codes	
Perception of care experience: Positive	Positive experience	1
Perception of care experience: Negative	Negative experience	6
Perception of care experience: Neutral or mixed	Neutral/mixed	4
Perception of care experience: Not clear	Unclear/not possible to discern OR no code assigned	5

The approach was not completely prescriptive, with several additional codes developed during the coding process to ensure the codebook captured the breadth of the data [36]. To ensure consistency of coding, coders first met to develop a shared theoretical understanding, interpretation and application of each code within the codebook before starting coding. To allow a closer reading of the data in relation to each topic, the codebook was split into three thematic framings: ‘Reflection on health care experiences’; ‘Description of healthcare experiences’; and ‘Mention of influences that related to or affected experiences with care’. A round of coding was undertaken for each theme. After independently coding each thematic section, coders came together to compare and compile codes. Any divergence in coding was resolved through discussion and often tended towards a consensus around one coders' interpretation. If consensus was not reached via this discursive approach, the open‐text response in question would be analysed by both coders, and a compromise reached that both were comfortable with [37]. For assessment of whether care received was adequate or inadequate, an absence of explicit mention of dissatisfaction or the need for further care in open‐text responses was ‘*inferred* as adequate’, while those where care was ‘*perceived* as inadequate’ were based on explicit mentions in the response. Assessment of ease of care access was based on inference from the coders based on the text, as well as explicit mentions by the respondent (see Table 5 quote 11 as an example where easy access was inferred, quote 5 as an example of explicit mention of difficult access).

#### 2.1.4 Socio‐Demographic, Health and COVID‐19 Illness Characteristics

Socio‐demographic characteristics were derived from self‐report at registration to the CSS app, CSSB consent or in the August 2022 CSSB questionnaire. Health characteristics were derived from self‐report at registration to the CSS app, CSSB consent, from a February 2021 CSS questionnaire, or from May 2021 and August 2022 CSSB questionnaires. COVID‐19 illness characteristics were derived from self‐report in the August 2022 questionnaire. Further details relating to data sources, question wording and processing prior to analysis are given in Supplementary Information Section S1.

### 2.2 Eligibility Criteria and Sample Selection

For all analyses, inclusion criteria were complete data on age, sex, ethnic group and area of residence.

For analysis of health and social care access issues during the COVID‐19 pandemic from closed question responses, individuals with partial completion of the 2022 CSSB questionnaire were excluded, giving an analysis sample of *N* = 3516. A full sample selection flow diagram is given in Figure S1.

For analysis of health care experiences for COVID‐19 from open text responses, sample selection was designed to allow the effect of multiple pre‐pandemic advantage/disadvantage to be estimated. To limit the number of open text responses to qualitatively code by hand to a manageable level, a subsample of all respondents with symptomatic SARS‐CoV‐2 infection and whose longest symptom duration COVID‐19 episode started at least 84 days before questionnaire completion was chosen. Prior to subsampling, participants were categorised into three socio‐demographic groups representing the accumulation of advantaged or disadvantaged statuses in the context of systems of social power and axes of inequity [38], named ‘relatively advantaged’, ‘intermediate’ and ‘relatively disadvantaged’. To generate socio‐demographic groups, categories of ethnic group, education level, local area deprivation, employment status and household income were first ascribed as representing relatively advantaged or disadvantaged statuses, as shown in Table 2. Socio‐demographic groups were then derived by categorisation of the number of advantages minus the number of disadvantages, as shown in Table 3. Participants with values of advantages–disadvantages = 0 and +2 were not included to create clear demarcations between groups. Finally, subsets of each socio‐demographic group were selected using stratified random sampling, with subsets proportionally representative in terms of age group, sex, long COVID status and self‐perceived COVID‐19 recovery status. The fraction of the socio‐demographic group selected for each subset was varied to give three subsets of approximately equal size. Aside from household income, which was collected in the 2022 questionnaire, other factors represent pre‐pandemic status. While gender was hypothesised as an additional axis of inequity, sex (as a proxy for gender) was not used to generate socio‐demographic groups, as it was thought more important that each group contained responses from female and male participants.

**Table 2:** Categories ascribed as representing relative advantage or disadvantage within systems of social power and axes of inequity, in the derivation of socio‐demographic groups representing accumulation of advantaged or disadvantaged statuses.

Factor	Advantaged status	Disadvantaged status
Ethnic group	White groups	Racially minoritised groups (Asian/Asian British, Black/Black British, mixed/multiple, any other)
Education level	Postgraduate level	Less than degree level
Local area deprivation	Low deprivation area (Index of Multiple Deprivation [IMD]) Decile 8–10)	High deprivation area (IMD Decile 1–3)
Pre‐pandemic employment status	—	Unemployed, long‐term sick or disabled
Annual gross household income	High (£100,000 or higher)	—

**Table 3:** Sample sizes and values for the number of advantages minus the number of disadvantages of socio‐demographic groups used in the analysis of open‐text questions on COVID‐19 health care experiences.

Socio‐demographic group	#Advantages–#disadvantages	Sample size of qualitatively coded subset
Relatively advantaged	+3, +4	109
Intermediate	+1	117
Relatively disadvantaged	−1, −2, −3	109

### 2.3 Statistical Analysis

#### 2.3.1 Regression Models and Proposed Causal Pathways

To obtain estimates of the effects of individual socio‐demographic variables on the number and likelihood of reporting health and social care access issues during the COVID‐19 pandemic, we used multivariable ordinary least squares (OLS) linear regression models (continuous outcome variable) and Poisson regression models with robust errors (binary outcome variable) [39]. Models used the ‘HC3’ estimator of coefficient standard errors to account for heteroskedasticity [40].

**Figure 1:**
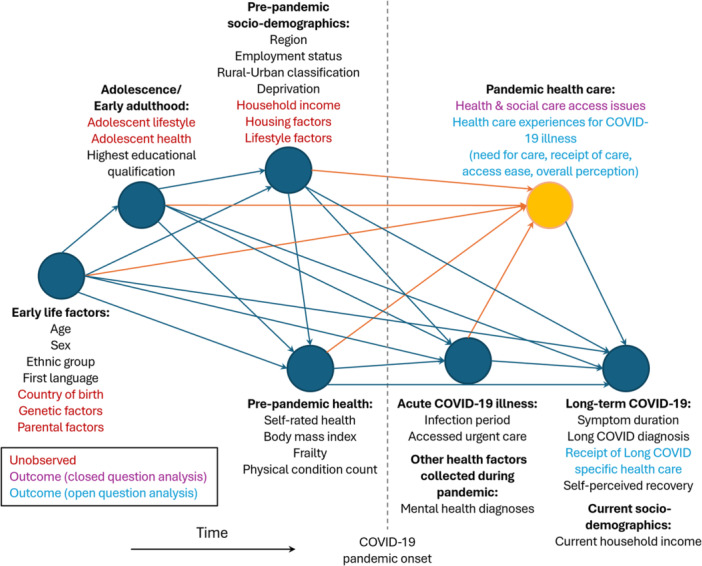
Directed acyclic graph describing hypothesised causal pathways. A proposed directed acyclic graph (DAG) is used to generate minimal adjustment variable sets for models estimating the association between exposure variables and outcomes related to health care during the COVID‐19 pandemic. Outcome variables derived from closed and open survey questions are highlighted in purple and blue, respectively, while key unobserved potential confounders are coloured in red. The DAG is structured in order of hypothesised data generation/crystallisation from left to right, and variables with similar time of data generation/crystallisation are grouped for clarity into ‘super nodes’. The pandemic health care super node and direct arcs to it are coloured in orange, to distinguish the node containing the majority of outcomes examined. The proposed DAG is ‘saturated’, in that each variable is hypothesised to be caused by all earlier variables.

For each exposure variable of interest, separate models were run including potential confounding variables as appropriate, based on the hypothesised directed acyclic graph (DAG), developed using DAGitty software: http://www.dagitty.net/dags.html (abridged DAG in Figure 1, full DAG in Figure S2, full DAGitty code given in Supporting Information) [41].

Associations between experiencing health and social care access issues and combinations of sex, education level and local area deprivation were estimated using mixed‐effects linear and logistic regression models, following the intersectional multilevel analysis of individual heterogeneity and discriminatory accuracy (MAIHDA) approach [42, 43], informed by the intersectionality framework [44, 45]. In models, a ‘social strata’ variable detailing the explicit combination of sex, education and deprivation acted as a level 2, random (intercept) effects variable, while the individual variables of sex, education and deprivation, plus age group and ethnic group as potential confounders, were included as level 1 fixed effects. Sex was used as a proxy for gender identity based on data availability. Sex, education and deprivation were chosen because of associations with sex and socio‐economic status previously observed in analyses of individual factors [1, 2], while also having strata sizes large enough to make reliable estimates.

For each social stratum, we present the stratum‐level average predicted number of access issues (from linear model) or the average predicted probability of one or more issues (from logistic model) generated from the fitted models, which capture both additive and interaction effects. The R script used to fit MAIHDA models was adapted from an MAIHDA tutorial [42, 46]; 95% confidence intervals for predicted probabilities are approximate only because the model assumes no sampling covariability between the regression coefficients and stratum random effects.

As supplementary and sensitivity analyses, additional MAIHDA models were run where: (1) the analysis sample was stratified by COVID‐19 history, to test how access to care varied within subsets of respondents with similar experiences of COVID‐19 illness; (2) household income, at the time of the 2022 questionnaire, was also included in the social strata variable, to further test the role of economic capital; (3) pre‐pandemic health factors were included as additional potential confounders, to test an alternative data generating timeline where health factors predominantly precede socio‐demographic factors.

To estimate the association between socio‐demographic group and aspects of COVID‐19 health care experiences, in an analysis of qualitatively coded open text responses, multivariable Poisson regression models were again used, with age group included as an adjustment variable.

#### 2.3.2 Generation of Inverse Probability Weights

Regression models included weights representing participants' inverse probability of questionnaire response and selection into the given analysis sample. Weights were used to reduce potential selection bias from differential response rates along socio‐demographic lines, as is often seen with volunteer cohorts [47], as well as collider bias from conditioning on COVID‐19 infection for analysis of COVID‐19 health care experiences. If infection is a mediating factor between exposures (pre‐pandemic socio‐demographics) and outcome (health care experiences), any unmeasured factor associated with both COVID‐19 infection and our outcome may bias estimated associations [48, 49]. Sets of weights were generated following a forward sequential feature selection method that optimised prediction (area under the receiver operating characteristic curve, AUC‐ROC, score) of presence in the analysis sample, described in more detail in previous CSSB studies [3, 50]. Weights were winsorised (bottom and top 5% values set equal to 5th and 95th percentile values), to limit the influence of individuals with extreme weight values, prior to use in regression models. Details of models used to generate inverse probability weights (IPWs) are summarised in Table S1.

#### 2.3.3 Software

Fixed‐effects regression models were fit, and figures were created, using Python v3.8.8 and packages: numpy v1.20.1, pandas v1.2.4, statsmodels v0.12.2, scipy v1.6.2, scikit‐learn v0.24.1, matplotlib v3.3.4 and seaborn v0.11.1. Intersectional MAIHDA mixed‐effects regression models were fit using R v4.3.0 and packages: haven v2.5.4, tidyverse v2.0.0, ggeffects v1.5.2, lme4 v1.1.35.2, merTools v0.6.2, labelled v2.13.0, sjPlot v2.8.16, Metrics v0.1.4 and dplyr v1.1.4.

### 2.4 Role of the Funding Source

The funders of the study had no role in the design of the study, data collection, data analysis, interpretation or writing of the report. All authors had full access to all data within the study. The corresponding authors had final responsibility for the decision to submit for publication.

## 3 Results

### 3.1 Part 1: Health and Social Care Access Issues During the COVID‐19 Pandemic (Closed Question Analysis)

#### 3.1.1 Sample Characteristics

Analysis of health and social care access issues during the COVID‐19 pandemic from closed questions was based on 3516 participants who completed the 2022 CSSB questionnaire (of 8324 invited, 42% response rate, sample selection shown in Figure S1). Questionnaire response was higher among older age groups and slightly higher among female participants, but not skewed by deprivation (Table S2). The median age group of respondents was 50–59 years old, most were female sex (81%), and identified as of white ethnic groups (97%) (Table 4). Respondents were relatively socio‐economically advantaged in comparison to the general UK population, tending to have high education and income levels, living in lower deprivation areas, and were mostly employed at the beginning of the COVID‐19 pandemic. Issues accessing health and social care were common, with 28% reporting one or more of the five issue types, and an average of 0.44 issues reported across all participants. The most common issue was accessing community‐based services (24%), and the least common was access to social care (2%). Among those reporting one or more care access issues, most experienced a single issue only (62%), with smaller proportions reporting two or more (38%), and three or more (12%) issues.

**Table 4:** Socio‐demographic characteristics and health and social care access issues. Unweighted group sizes, unadjusted proportions of participants with one or more health and social care access issues during the COVID‐19 pandemic, and mean number of issues, split by participant socio‐demographics. Socio‐demographic categories acting as references in multivariable regression models are indicated. Figures for group sizes of less than 5 are suppressed.

		Group size		One or more health and social care access issues		Mean number of issues
Domain	Variable	*N*	%	*N*	%	
	**Total**	3516		999	28.4%	0.44
Health and social care access issues during the COVID‐19 pandemic	**Health and social care access issue type**					
	Unable to access required medication	144	4.1%			
	Unable to access health services in the community	827	23.5%			
	Unable to access the community social care services or voluntary sector support needed	73	2.1%			
	Unable to access inpatient or outpatient appointments booked at a hospital	388	11.0%			
	Unable to access appointment for cognitive behaviour therapy, counselling, or psychological therapy	127	3.6%			
	**Health and social care access issue count**					
	None	2517	71.6%			
	One	624	17.7%			
	Two	251	7.1%			
	Three	81	2.3%			
	Four	25	0.7%			
	Five	18	0.5%			
Individual pre‐pandemic demographics	**Age (median, interquartile range)**	59 (52–65)		58 (52–65)		
	**Age group (years)**					
	18–39	186	5.3%	47	25.3%	0.51
	40–49	493	14.0%	148	30.0%	0.47
	50–59 (reference)	1144	32.5%	362	31.6%	0.51
	60–69	1254	35.7%	322	25.7%	0.37
	≥ 70	439	12.5%	120	27.3%	0.40
	**Sex**					
	Female (reference)	2840	80.8%	843	29.7%	0.47
	Male	676	19.2%	156	23.1%	0.32
	**Ethnic group**					
	Asian/Asian British	18	0.5%	< 5	—	0.33
	Black/Black British	15	0.4%	< 5	—	0.27
	Mixed/Multiple	29	0.8%	9	31.0%	0.45
	Other	38	1.1%	11	28.9%	0.42
	White (reference)	3416	97.2%	972	28.5%	0.44
	**First language**					
	English (reference)	3437	97.8%	976	28.4%	0.44
	Other	76	2.2%	23	30.3%	0.50
	Prefer not to answer/not stated	<5	—	<5	—	—
	**Highest educational qualification**					
	Prefer not to answer/not stated	40	1.1%	13	32.5%	0.58
	Did not complete secondary school	25	0.7%	< 5	—	0.28
	GCSE or GNVQ or equivalent	412	11.7%	139	33.7%	0.52
	A‐Levels or advanced GNVQ or equivalent	587	16.7%	183	31.2%	0.45
	University degree (reference)	1269	36.1%	341	26.9%	0.44
	Postgraduate degree or higher	1014	28.8%	272	26.8%	0.42
	PhD	169	4.8%	47	27.8%	0.43
	**UK region**					
	East Midlands	227	6.5%	74	32.6%	0.56
	East of England	377	10.7%	113	30.0%	0.49
	London (reference)	593	16.9%	163	27.5%	0.45
	North East	109	3.1%	29	26.6%	0.41
	North West	342	9.7%	95	27.8%	0.40
	Scotland and Northern Ireland	178	5.1%	54	30.3%	0.50
	South East	693	19.7%	195	28.1%	0.44
	South West	360	10.2%	90	25.0%	0.41
	Wales	180	5.1%	53	29.4%	0.44
	West Midlands	223	6.3%	67	30.0%	0.44
	Yorkshire and The Humber	234	6.7%	66	28.2%	0.37
	**Pre‐pandemic employment status**					
	Employed (reference)	1777	50.5%	530	29.8%	0.45
	Self‐employed	390	11.1%	106	27.2%	0.42
	Unemployed	15	0.4%	6	40.0%	0.87
	Permanently or long‐term sick or disabled	46	1.3%	35	76.1%	1.83
	Retired	928	26.4%	206	22.2%	0.32
	Other	287	8.2%	87	30.3%	0.48
	Unknown	73	2.1%	29	39.7%	0.78
	**Rural–Urban classification**					
	Rural	864	24.6%	227	26.3%	0.39
	Urban (reference)	2652	75.4%	772	29.1%	0.46
	**Local area deprivation**					
	IMD Decile 1 (most deprived 10%)	82	2.3%	37	45.1%	0.73
	IMD Decile 2	129	3.7%	42	32.6%	0.52
	IMD Decile 3	185	5.3%	57	30.8%	0.50
	IMD Decile 4	255	7.3%	90	35.3%	0.58
	IMD Decile 5	314	8.9%	95	30.3%	0.49
	IMD Decile 6	406	11.5%	122	30.0%	0.48
	IMD Decile 7	428	12.2%	129	30.1%	0.48
	IMD Decile 8	495	14.1%	131	26.5%	0.41
	IMD Decile 9	563	16.0%	150	26.6%	0.41
	IMD Decile 10 (least deprived 10%) (reference)	659	18.7%	146	22.2%	0.31
Socio‐demographics during the pandemic	**Current household income**					
	Prefer not to answer/not stated	467	13.3%	146	31.3%	0.49
	Less than £20,000	318	9.0%	127	39.9%	0.68
	£20,000–£29,999	348	9.9%	103	29.6%	0.47
	£30,000–£39,999	425	12.1%	128	30.1%	0.48
	£40,000–£49,999	415	11.8%	111	26.7%	0.46
	£50,000–£74,999 (reference)	649	18.5%	178	27.4%	0.40
	£75,000–£99,999	378	10.8%	106	28.0%	0.41
	£100,000 or more	516	14.7%	100	19.4%	0.28

Just over half of respondents reported ‘excellent’ or ‘very good’ general health at the beginning of the COVID‐19 pandemic (55%, Table S3). The proportion of respondents with a history of COVID‐19 illness (83%) and prior or ongoing long COVID (22% with prior or ongoing self‐reported long COVID, 9% with diagnosed long COVID and 25% with ongoing symptoms at the time of the questionnaire) was higher than the general UK population at the time of questionnaire invitation, reflecting targeted recruitment into CSSB. The vast majority of self‐reported COVID‐19 illness was community‐based (8% of those with COVID‐19 history accessed urgent care during illness), and confirmed by self‐reported positive antibody or antigen tests (88% of those with COVID‐19 history).

#### 3.1.2 Associations Between Individual Socio‐Demographic Factors and Health and Social Care Access Issues

**Figure 2:**
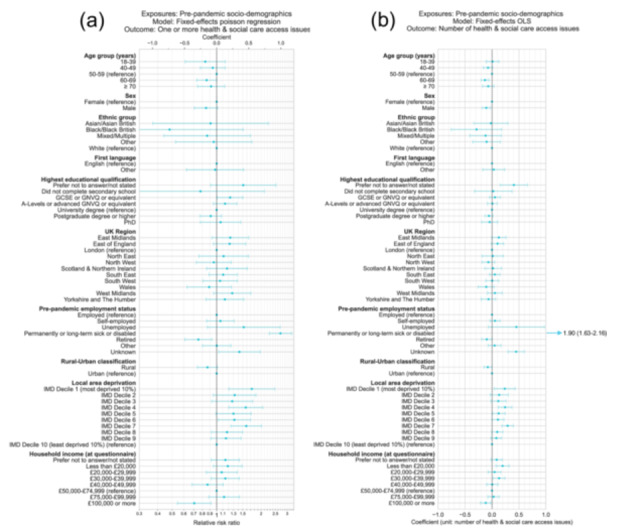
Coefficients and 95% confidence intervals from (a) Poisson regression and (b) ordinary least squares linear regression models estimating associations between pre‐pandemic socio‐demographic factors and health and social care access issues during the COVID‐19 pandemic. Results for each exposure variable originate from models with distinct adjustment variable sets to control for potential confounding based on hypothesised DAG as follows: Age: [Sex]; Sex: [Age]; Ethnic group: [Age]; First language: [Age, Sex, Ethnic group]; Education: [Age, Sex, Ethnic group, First language]; Region: [Age, Sex, Ethnic group, First language, Education]; Pre‐pandemic employment status: [Age, Sex, Ethnic group, First language, Education, Region]; Rural–Urban classification (RUC): [Age, Sex, Ethnic group, First language, Education, Region, Pre‐pandemic employment status]; Local area deprivation: [Age, Sex, Ethnic group, First language, Education, Region, Pre‐pandemic employment status, RUC]; Household income (at time of questionnaire): [Age, Sex, Ethnicity, First language, Education, Region, Pre‐pandemic employment status, RUC, Local area deprivation, Pre‐pandemic health factors, COVID‐19 illness factors]. Models included participant weighting for inverse probability of questionnaire completion.

The effects of individual pre‐pandemic socio‐demographic variables on health and social care access during the COVID‐19 pandemic were estimated, using both linear regression to estimate the count of health and social care access issues (of 5) as a continuous outcome variable, and modified Poisson regression to estimate the likelihood of one or more health care access (Figure 2; Table S3). Models controlled for potential confounding based on hypothesised DAG and used inverse probability weights to account for the probability of questionnaire completion.

Largest effects were observed for pre‐pandemic employment status, where those long‐term sick or disabled reported a much higher average number of care access issues versus employed participants (OLS coefficient: 1.90, 95% CI: 1.63–2.16; Poisson regression RR: 2.70, 95% CI: 2.27–3.21).

Effects were also observed in both linear and Poisson regression models for those living in more deprived areas, who were more likely to report issues versus the least deprived 10% of areas (most deprived 10%, OLS: 0.24, 0.04–0.43; RR: 1.73, 1.21–2.47). Fewer issues were also observed for those with household income of more than £100,000 at the time of the questionnaire versus £50,000–74,999 (OLS: −0.12, −0.21 to −0.02; RR: 0.71, 0.54–0.92), and for those aged 60–69 versus 50–59 (with no other clear trend with age) (OLS: −0.13, −0.20 to −0.06; RR: 0.86, 0.73–1.00).

Lower number of access issues were also observed, but in linear regression models only, and with smaller effect sizes, for male versus female participants, those living in rural versus urban areas, for those retired versus employed at the start of the COVID‐19 pandemic, and for those with undergraduate degree level versus those who preferred not to disclose their education level. Finally, increased likelihood of access issues was also observed, but in Poisson regression models only, for participants with GCSE or GNVQ or equivalent (typically finishing education at age 16) versus undergraduate degree level.

#### 3.1.3 Intersectional MAIHDA Analysis of Inequalities in Health and Social Care Access Issues

3.1.4

**Figure 3:**
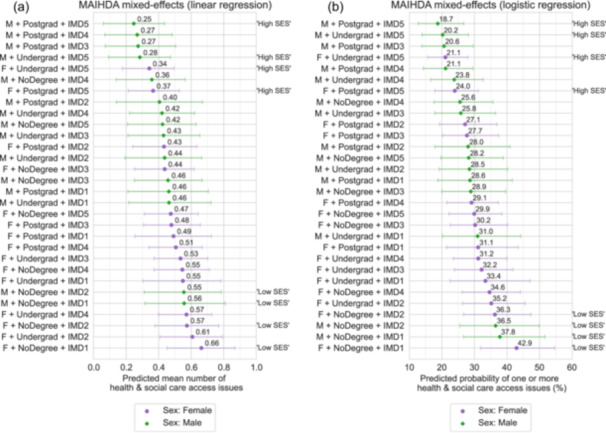
Intersectional MAIHDA models estimating the effect of combinations of sex, education level and local area deprivation on health and social care access issues during the COVID‐19 pandemic. (a) Predicted mean (with approximate 95% confidence intervals) number of issues from linear regression and (b) predicted probability (with approximate 95% confidence intervals) of one or more issues from logistic regression. Fixed effects included in models were age group, ethnic group, sex, education level and local area deprivation. Models included participant weighting for inverse probability of questionnaire completion. Strata labels: F = Female, M = Male; NoDegree = Less than degree level education (including not stated/prefer not to say), Undergrad = Undergraduate degree level, Postgrad = Postgraduate degree level or higher; IMD1‐IMD5 = Index of Multiple Deprivation Quintile 1 to 5, where 1 is the most deprived 20% of areas, and 5 is the least deprived 20%. Labels ‘High SES’ are given to highlight strata where the combination of education level and deprivation suggests relatively high socio‐economic status—living in the least deprived quintile 1 and having a degree level education or higher. ‘Low SES’ highlights relatively low socio‐economic status strata—living in the most deprived 40% (quintile 4 or 5) and having less than a degree‐level education.

In addition to estimating associations between individual pre‐pandemic socio‐demographic variables and health and social care access during the COVID‐19 pandemic, intersectional MAIHDA analysis was performed to estimate associations between ‘social strata’, with particular combinations of sex, education level and local area deprivation, and care access, while taking into account interaction effects (Figure 3; Tables S4 and S5).

MAIHDA intersectional models found large variation in health and social care issues between strata, with the predicted mean number of issues varying between 0.25 (approximate 95% CI: 0.06–0.44) and 0.66 (0.45–0.87), and the likelihood of one or more issues varying between 18.7% (12.8%–26.7%) and 42.9% (31.9%–54.6%). Female sex strata tended to have a higher probability of having one or more health and social care issues, and a higher predicted mean number of issues, than male strata. However, male as well as female sex strata with socio‐economically disadvantaged combinations of both education and local area deprivation—that is, low education levels and living in high deprivation areas (‘Low socio‐economic status [SES]’ strata)—were consistently the most likely to report health and social care access issues. In contrast, both female and male sex strata with high education levels living in low deprivation areas (‘High SES’) were among those least likely to report issues.

The variance partition coefficients (VPC) of MAIHDA models were low (VPC = 0.96% for both linear and logistic regression models), indicating that the vast majority of the variation in care access issues between strata is accounted for by additive effects of individual variables, with interaction effects making a relatively small contribution.

In supplementary analysis, additional MAIHDA models were run on samples stratified by COVID‐19 history (Figure S3). The size of the inequality in care access issues between strata was slightly larger for the ‘no COVID’ and ‘long COVID’ subsamples, compared to the ‘short COVID’ subsample. Similar to the overall sample, the likelihood of care access issues was higher for female strata and ordered along the lines of socio‐economic status in all stratified subsamples, albeit with small differences in the relative importance of deprivation and education.

To further evaluate the role of economic capital on access to care, household income was also included in the social strata variable (Figure S4). It is noted that data represented income at the time of the questionnaire, and so may be subject to reverse causality if health care access issues during the pandemic caused declines in income. Nevertheless, after including income, strata with the highest predicted probability of health and social care access issues were consistently those with the lowest income band (< £20,000), in combination with fewer educational qualifications and/or living in higher deprivation areas, while strata with lowest predicted probability were those with the highest income band (≥ £100,000) in combination with higher education level and/or living in lower deprivation areas. The range of the inequality across strata was also increased with the addition of income for both linear regression (range = 0.71 vs. 0.41) and logistic (range = 34% vs. 24%) models.

In final sensitivity analyses, negligible changes were observed in MAIHDA model predictions after additional adjustment for pre‐pandemic health factors (themselves tested for associations, with results presented in Table S3), representing an alternative hypothesised causal diagram where health precedes social factors (vs. social factors preceding health factors that is assumed in the main analysis) (Figure S5).

### 3.2 Part 2: Health Care Experiences for COVID‐19 (Open Question Analysis)

#### 3.2.1 COVID‐19 Health Care Experiences From Qualitative Coding

To explore health care experiences specifically for COVID‐19 illness in more detail, free‐text responses to an open survey question on health care experiences for COVID‐19 were coded using a deductive approach for a subset of respondents (*N* = 335) (outcomes given in Table 1, full codes and frequencies given in Table S6). The subset was comprised of three approximately equal‐sized groups selected to assess experiences of respondents with varying degrees of advantaged or disadvantaged positions/statuses/characteristics (selection criteria are given in Tables 2 and 3, socio‐demographic characteristics of the subset are given in Table S7).

**Figure 4:**
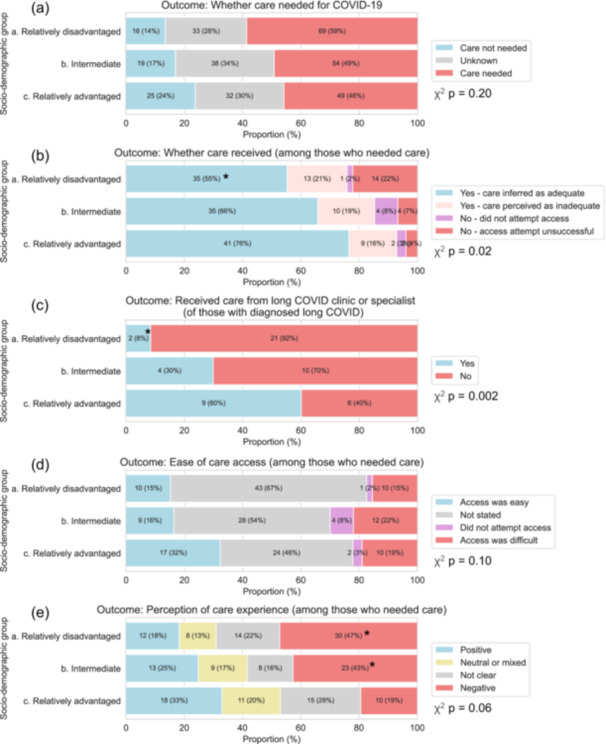
Frequency of various COVID‐19 health care experiences from qualitative coding of free‐text responses. Bar labels present pseudo‐population sample sizes and proportions after weighting for the inverse probability of selection into the qualitatively coded subset (a) and needing care for COVID‐19 (b–e). Bars with * highlight where differences in comparison with the relatively advantaged group were statistically significant, *p* < 0.05, from multivariable Poisson regression models adjusting for age group (presented in Figure S6). *p*‐values for chi‐squared tests of independence are also presented.

Quantitative analysis of qualitatively coded open text responses found socio‐demographic gradients across multiple aspects of COVID‐19 health care experiences (Figure 4, with illustrative quotes given in Table 5). Despite the small sample size of the qualitatively coded subset, multiple observations of poorer care access and experiences for more disadvantaged versus more advantaged participant groups were statistically significant when tested in multivariable Poisson regression models adjusting for age group (Figure S6). These observations were: lower likelihood of feeling that they have received adequate care, among those who expressed need for care (RR = 0.69, 95% CI: 0.50–0.96, *p* = 0.03 for the relatively disadvantaged group vs. the relatively advantaged group from multivariable regression models adjusting for age group); lower likelihood of receiving specialist care for long COVID among those with a long COVID diagnosis (RR = 0.14, 95% CI: 0.04–0.50, *p* = 0.003 for relatively disadvantaged vs. relatively advantaged); and higher likelihood of negative experiences (RR = 2.44, 95% CI: 1.24–4.80, *p* = 0.01 for relatively disadvantaged vs. relatively advantaged). Also of note, but not significant at a *p* < 0.05 level, were apparent higher proportions of relatively disadvantaged participants reporting unsuccessful attempts to obtain care, and easier access to care among relatively advantaged participants.

**Table 5:** Illustrative quotes from free‐text responses describing COVID‐19 health care experiences, including the health care outcomes ascribed to the quotes, the socio‐demographic group used in quantitative analysis, sex and age group. The first quote that includes a certain type of experience is highlighted in bold. Potentially identifying text is masked, and spelling is occasionally edited for clarity. Quotes are otherwise reproduced verbatim.

#	Outcome	Socio‐demographics	Quote
1	**Care needed for COVID‐19: No**	Relatively advantaged, Female, 40–49	I did not need to contact my GP about my covid infection
2	**Care needed for COVID‐19: Unknown**	Intermediate, Female, 60–69	I self‐isolated and diagnosed myself. I had general medication at home, which I used
3	**Care needed for COVID‐19: Yes** **Care received: No, did not attempt to access**	Intermediate, Male, 70+	Did not bother because I had no chance of seeing a GP
4	**Care received: No, access attempt unsuccessful** **Perception of care experience: Negative** **Ease of care access: Not stated**	Relatively disadvantaged, Female, 50–59	I still can't get to see GP. I don't think I'm [ill] enough to warrant an appointment. I have avoided hassling them as I know they are under a great deal of pressure
5	**Care received: Yes, perceived as inadequate** Perception of care experience: Negative **Ease of care access: Difficult**	Relatively disadvantaged, Female, 50–59	It was really hard to see GP and still is. I am still waiting for a specialist lung appointment, and I [have] a nodule on my lung, my cough is awful, and I am always tired. The health care is rubbish since COVID.
6	Care received: Yes, perceived as inadequate Perception of care experience: Negative Ease of care access: Difficult **Received care from a long COVID clinic or specialist**	Relatively disadvantaged, Female, 50‐59	I found the whole process of trying to get any help and support [from] either the GP surgeries and or 111 or the hospitals extremely stressful and difficult! Apart from being very unwell and unable to do the most basic of things, I was left with the awful task of seeking help myself and, in most cases, even begging for help! Trying to get an appointment just to speak to a GP was near enough impossible. I also found that the surgeries and the [GPs] were not informed or knowledgeable enough to even really give any right advice. They were not well informed of any of the Long‐Covid Clinics available. [In fact] I had to tell them about the availability of such clinics!! They even asked ME to find out about them and pass on these details to THEM!! When clearly it should [in fact] be THEIR duty to help and aid their patients! Right? Even after me finding these Clinics, there was so much red tape to deal with! I was told I was not allowed to be referred to the [CITY NAME] Clinics, e.g., the [HOSPITAL NAME] one or the [HOSPITAL NAME]. Then I was told on a number of times that I had [been put] on a waiting list, when [in fact] I hadn't been! Sheer gross negligence, I would say, on their part. This meant that I was [extremely] delayed in getting any care from the Long‐Covid Clinic at all.
7	Care received: Yes, perceived as inadequate Perception of care experience: Negative Ease of care access: Difficult	Relatively disadvantaged, Female, 70+	I got no support the first time I had COVID. I had a phone call consultation with my doctor on my mobile when I was walking along the street. Long Covid made it difficult for me to marshal my thoughts, and my symptoms kept changing. I felt desperate but had no support from the doctor. I felt as though I was being viewed as a hypochondriac. I had a couple of phone consultations, but never saw a doctor. Eventually, I was referred to a chest specialist for my cough she diagnosed gastric reflux. The second time I had Covid, I was not so [ill], and I didn't consult my GP
8	**Care received: Yes, inferred as adequate** **Perception of care experience: Positive** **Ease of care access: Easy**	Intermediate, Female, 60–69	Accessed out of hours for breathlessness. Seen very quickly and received excellent care, checked my pO2 and prescribed steroids. Breathlessness resolved.
9	Care received: Yes, inferred as adequate Perception of care experience: Positive Ease of care access: Easy Received care from a long COVID clinic or specialist	Relatively advantaged, Female, 50‐59	GP was very helpful, supportive and understanding when I had long COVID in the first half of 2021. All over the phone, of course, but it still worked well. It was early days in terms of NHS support for long COVID, but the GP surgery tracked down the Yorkshire Rehabilitation Protocol (?) forms and submitted them into the system, and I then had several phone calls in the following few months from a long COVID clinic. I had mostly recovered by then, but it was really reassuring to know someone was there, and it helped to speak to them. The app was launched not long after, and it also had good information in it. I was impressed with what the NHS provided, overall.
10	Care received: Yes, inferred as adequate Perception of care experience: Positive Ease of care access: Easy Received care from a long COVID clinic or specialist	Relatively advantaged, Male, 40–49	I asked my GP for a referral to the long COVID service, and she did so after checking my symptoms. I saw a consultant and physio together (MDT). They gave me some advice and referred me to a novel treatment—hydrotherapy in a Dead Sea salt therapy pool. The pool sessions were useful, helpful and enjoyable. I was put onto PIFU, and I re‐contacted them when my symptoms flared up again. I self‐referred to talking therapies. I have learned that long COVID has significantly changed who I am—physically AND mentally. My former self‐image of a healthy, active, sporty man has been replaced by a tired, depressed and unwell person. This has had a significant impact on my mental health.
11	Care received: Yes, inferred as adequate Perception of care experience: Positive Ease of care access: Easy	Relatively disadvantaged, Female, 40–49	Was able to speak to a nurse on the phone who happily prescribed whatever asthma meds and steroids to help my breathing—weren't testing generally then, so couldn't tell if it was COVID or not. Advice on 111 was very helpful, e.g., pulseoximetry reading help, and when to call 999
12	Care received: Yes, inferred as adequate Perception of care experience: Negative Ease of care access: Difficult Received care from a long COVID clinic or specialist	Intermediate, Male, 50–59	Long time (months) to see someone at a Long Covid clinic, clinic referred me to other clinics to help rule other things out, but hard to contact or arrange an appointment with lots of different services.
13	Care received: Yes, perceived as inadequate **Perception of care experience: Neutral or mixed** Ease of care access: Difficult	Relatively disadvantaged, Female, 60–69	Almost impossible to see the GP. I felt so poorly for weeks after. GP did arrange for a blood test, and as that was ‘normal’, no further support was available. My local pharmacist was amazing. Gave advice over the telephone whenever I called.
14	Care received: Yes, perceived as inadequate Perception of care experience: Neutral or mixed Ease of care access: Not stated Received care from a long COVID clinic or specialist	Intermediate, Female, 18–39	GPs seemed to have limited knowledge of the Long Covid condition, even 18 months into the pandemic. Switching to the GP telephone triage appointment system was fine for me, and I could access my prescriptions online. Long Covid clinics have been helpful with more understanding, referrals and tests, but without a concrete condition to treat and discharge, I have reached the limit of what help they can offer me and am at a loss of what to do next.
15	Care received: Yes, inferred as adequate Perception of care experience: Neutral or mixed Ease of care access: Difficult	Relatively advantaged, Female, 70+	It was hard to have contact with NHS GPs and Doctors. So I resorted to the private system using [PRIVATE PROVIDER NAME], and it was much better. A private GP helped me.
16	Care received: Yes, inferred as adequate **Perception of care experience: Not clear** Ease of care access: Difficult	Relatively disadvantaged, Female, 60–69	I've been told that had it not been for my tenacious attitude, I wouldn't have had the many tests I have undergone. I have had three telephone consultations with a Respiratory Consultant and several telephone consultations with a respiratory physiotherapist, and have now been referred to a Breathing Clinic where I am currently undergoing lung review tests, etc. I was originally diagnosed with bronchiectasis, but this has now been thankfully downgraded to Long Covid.

The contrasts in experiences between participants with different socio‐economic positions are exemplified by comparing illustrative quotes 9 and 5 from Table 5:

‘GP was very helpful, supportive and understanding when I had long covid in the first half of 2021. All over the phone of course but it still worked well. It was early days in terms of NHS support for long covid but the GP surgery tracked down the Yorkshire Rehabilitation Protocol (?) forms and submitted them into the system and I then had several phonecalls in the following few months from a long covid clinic. I had mostly recovered by then but it was really reassuring to know someone was there and it helped to speak to them. The app was launched not long after and also had good information in it. I was impressed at what the NHS provided, overall’.

Quote 9. Relatively advantaged, Female, 50–59.

‘It was really hard to see GP and still is. I am still waiting for specialist lung appointment and I [have] a nodule on my lung, my cough is awful and I am always tired. The health care is rubbish since covid’.

Quote 5. Relatively disadvantaged, Female, 50–59.

Both quotes were made by female participants of white ethnic groups in their 50s, both living in the same region of England, and both with diagnoses of long COVID. However, the participant who wrote quote 9 was part of the relatively advantaged group, by virtue of having a postgraduate level of education, being in the highest income bracket (≥ £100,000), and living in an area among the least deprived 30% in the United Kingdom. In contrast, quote 5's participant was part of the relatively disadvantaged group, with a less than degree level of education, below the median level of household income (< £40,000), and lived in an area among the most deprived 30% in the United Kingdom.

Unsuccessful attempts to access care and negative experiences most often concerned not being able to get appointments with doctors in primary care. Several responses also described having long waiting times for long COVID clinics, and difficulties obtaining relevant treatment from GPs (e.g., Table 5, quotes 4, 5 and 6). Care ascribed as inadequate often described feeling unsupported by health professionals and having a lack of treatment options (e.g., Table 5, quotes 6, 7 and 14). In contrast, positive experiences often described fast and easy access to care, referrals to specialist services and receipt of multiple treatments (e.g., Table 5, quote 10). Almost all positive experiences among relatively advantaged participants mentioned their GP (e.g., Table 5, quote 10), while relatively disadvantaged participants mentioned using other services such as pharmacy and 111 or 999 more frequently (e.g., Table 5, quote 11).

## 4 Discussion

### 4.1 Key Points

Analysis of both closed and open survey questions emphasised the cumulative effects of holding multiple socially advantaged or disadvantaged positions on health care access and experiences among participants of the COVID Symptom Study Biobank United Kingdom longitudinal population study.

In the first part of our study, analysis of a closed survey question from participants of the COVID Symptom Study Biobank UK longitudinal population study found large socio‐demographic inequalities in the frequency of issues accessing health and social care in the first two and a half years of the COVID‐19 pandemic in the United Kingdom. Gradients were found along the lines of social power and socio‐economic status when looking at variation based on explicit combinations of sex, education, local area deprivation and income, with more advantaged participants less likely to experience issues accessing care (Figure 3). Female strata consistently reported more care access issues, while the highest and lowest probabilities of issues were seen for the most socio‐economically advantaged or disadvantaged strata among both females and males. Findings are consistent with previous UK studies of health care disruption during the COVID‐19 pandemic, which found poorer access for females [1, 2], and those in lower income occupational classes [1], as well as trends of poorer access to health care for lower income groups across the European Union [51]. Associations were not explained by pre‐pandemic health (Figure S5), and similar socio‐economic gradients were also observed within groups with comparable histories of COVID‐19 illness (Figure S3), suggesting that social inequalities in access to care exist independent of the specific illnesses for which care is needed.

Similar social gradients were then observed in quantitative analysis of a subset of deductively coded free‐text responses to an open question about health care experiences for COVID‐19 illness (Figure 4). Analysis suggested less frequent access to adequate care and a higher frequency of negative experiences for more disadvantaged participants. Receipt of long COVID specialist care was also socially patterned, even among those who had a long COVID diagnosis. Multiple observations were statistically significant despite the subset's small sample size. Illustrative quotes provided detailed accounts of the multiple aspects of care that were found to vary with social circumstances (Table 5).

### 4.2 Interpretation

Observed inequalities in health care may be explained both in terms of structural differences at an area level, and individual level differences in social, cultural and economic capitals, following Bourdieu's conceptualisation of social class [52], as well as the related concept of health capital [53].

At a structural level, lower funding and workforce provision in primary health care services in more deprived areas [54, 55] are likely to contribute to inequalities we observe, perhaps most significantly to initial access to care and waiting times, as well as poorer interpersonal experiences due to greater time constraints. While higher personal income may provide the financial means to access care privately, differences in other forms of individual capital may explain more of the observed inequalities in care experiences beyond initial access. Higher cultural or health capital, such as greater education or understanding of health and health care services, may help more advantaged patients to self‐advocate at a time when persistence appears to be key [25]. Meanwhile, social capital, in the form of knowing health care professionals, may aid navigation through a complex and fragmented health care system. Stark contrasts in interpersonal experiences with health care professionals (mostly GPs) between advantaged and disadvantaged participants also echo well‐known social gradients in patient–doctor communication favouring higher socio‐economic status patients [56], and may be explained in part by bias from health care providers against lower socio‐economic status patients, which appears to negatively affect clinical decision making [57].

Considering health care specifically for long COVID, the poorer access to both general and specialist long COVID health care for less advantaged participants is further evidence of an inverse care law in action. Less frequent receipt of specialist care despite having a long COVID diagnosis was particularly concerning (Figure 4c). Furthermore, the repeated description by participants living with long COVID of feeling like they had no treatment options emphasises the need for continued support for patients as well as the need for further research to build upon recent trials of rehabilitation programmes [34], and improve the efficacy of the UK post‐COVID clinics [58].

### 4.3 Limitations

We note the limitations in our study. Recruitment into the CSSB was conditioned on the use of a smartphone app and targeted based on COVID‐19 histories. As such, the cohort over‐represents experiences of people with a history of long COVID, as well as female sex, white ethnic groups and more socio‐economically advantaged groups compared to the UK population, and so results may not be generalisable to the United Kingdom as a whole.

Our analysis samples were subject to CSSB members voluntary participation in surveys, as well as selection of subsets, which may be subject to selection bias, with higher participation among older age groups and female participants (Table S2). We attempted to mitigate bias by using inverse probability weighting, with a dedicated set of weights generated for each analysis subset.

Choice of factors to examine in intersectional MAIHDA models was limited by sample size; for example, inclusion of ethnic groups was not feasible due to small numbers of racially minoritised ethnic groups. Income data represented current income at the time of the 2022 questionnaire, rather than prior to the COVID‐19 pandemic, and so may be subject to reverse causality if health care access issues during the pandemic caused declines in income. As such, income was only included in supplementary analysis models. Similarly, the number of open‐text responses able to be coded by hand was limited, and so we were not able to analyse explicit intersectional inequalities in COVID‐19 using an approach such as MAIHDA.

### 4.4 Summary

In summary, our study suggests there were large intersectional inequalities in health care access and experiences during the COVID‐19 pandemic in the United Kingdom, for people with or without a history of COVID‐19 illness, along the lines of structural societal advantage. Further research could focus on identifying how much of the observed inequalities in health care quality arise due to differences in interpersonal versus structural factors, in order to address issues in both initial access to health care services and experiences upon entry. Finally, further evidence of greater unmet need for care among disadvantaged participants living with long COVID emphasises the continued need for support that works for those with the least power and status within society.

## Author Contributions

**Nathan J. Cheetham:** conceptualisation, methodology, formal analysis, investigation, visualisation, data curation, writing – review and editing, writing – original draft. **Anoushka Beattie:** conceptualisation, methodology, formal analysis, data curation, writing – review and editing. **Alastair B. Comery:** conceptualisation, methodology, formal analysis, data curation, writing – review and editing. **Vicky Bowyer:** conceptualisation, project administration, investigation, data curation, writing – review and editing. **J. D. Carpentieri:** conceptualisation, methodology, writing – review and editing. **Claire J. Steves:** conceptualisation, funding acquisition, project administration, methodology, writing – review and editing.

## Ethics Statement

Yorkshire and Humber NHS Research Ethics Committee gave ethical approval for the COVID Symptom Study Biobank, Ref: 20/YH/0298.

## Conflicts of Interest

The authors declare no conflicts of interest.

## Supporting information

Supporting File

## Data Availability

For the purposes of open access, the author has applied a Creative Commons Attribution (CC BY) licence to any Accepted Author Manuscript version arising from this submission. Access to data in the COVID Symptom Study Biobank is available to bona fide health researchers on application to the COVID Symptom Study Biobank Management Group. Further details are available online at https://cssbiobank.com/information-for-researchers, including application forms and contact information. The analysis code used in this study is available openly on GitHub at https://github.com/nathan-cheetham/CSSBiobank_CareExperiences. Anonymised COVID Symptom Study data are available to researchers to be shared with researchers according to their protocols in the public interest through Health Data Research UK (HDRUK) and Secure Anonymised Information Linkage consortium, housed in the UK Secure Research Platform (Swansea, UK) at https://web.www.healthdatagateway.org/dataset/fddcb382-3051-4394-8436-b92295f14259.
